# *Salicornia dolichostachya* organosolv fractionation: towards establishing a halophyte biorefinery

**DOI:** 10.1039/d2ra04432c

**Published:** 2022-10-07

**Authors:** Maxwel Monção, Tobias Wretborn, Ulrika Rova, Leonidas Matsakas, Paul Christakopoulos

**Affiliations:** Department of Civil, Environmental and Natural Resources Engineering, Luleå Tekniska Universitet SE-971 87 Luleå Sweden leonidas.matsakas@ltu.se +46 (0) 920 493043

## Abstract

Halophytes are a potential source of lignocellulosic material for biorefinery, as they can be grown in areas unsuitable for the cultivation of crops aimed at food production. To enable the viable use of halophytes in biorefineries, the present study investigated how different organosolv process parameters affected the fractionation of green pressed fibers of *Salicornia dolichostachya*. We produced pretreated solids characterized by up to 51.3% ± 1.7% cellulose, a significant increase from 25.6% ± 1.3% in untreated fibers. A delignification yield of as high as 60.7%, and hemicellulose removal of as high as 86.1% were also achieved in the current study. The obtained cellulose could be completely converted to glucose *via* enzymatic hydrolysis within 24 h. The lignin fractions obtained were of high purity, with sugar contamination of only 1.22% w/w and ashes below 1% w/w in most samples. Finally, up to 29.1% ± 0.4% hemicellulose was recovered as a separate product, whose proportion of oligomers to total sugars was 69.9% ± 3.0%. To the best of our knowledge, this is the first report in which *Salicornia* fibers are shown to be a suitable feedstock for organosolv biomass fractionation. These results expand the portfolio of biomass sources for biorefinery applications.

## Abstract 2

An organosolv method was developed for the fractionation of fibers of a halophyte plant in a biorefinery approach. *Salicornia dolichostachya* was used as raw material allowing the production of cellulose, hemicellulose, and lignin fractions.

## Graphical abstract

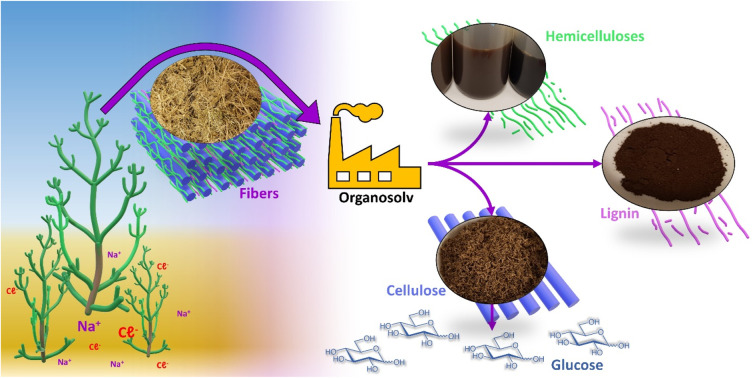

## 1. Introduction

The increasingly limited availability of non-renewable energy sources and raw materials requires a more sustainable use of natural resources and, consequently, better understanding of upcycling processes.^1,2^ The use of lignocellulosic biomass in biorefinery represents a sustainable alternative to fossil resources for the production of chemicals and energy. Biomass is composed primarily of carbohydrates, including cellulose, hemicellulose, and lignin, as well as varying amounts of extractives. Hence, biomass fractionation is of paramount importance for a holistic biomass use.^3–5^ In particular, successful fractionation of lignocellulosic substrates enables the valorization of all biomass components for their use in the manufacturing of biofuels, prebiotics, pharmaceuticals, chemicals, and cosmetics.^6–8^

Organosolv fractionation has attracted increasing interest owning to its ability to separate lignocellulosic biomass into high-quality cellulose, hemicellulose, and lignin streams.^9,10^ During organosolv, lignocellulosic biomass is treated at high temperatures with different combinations of solvents (*e.g.*, ethanol) and water. The resulting fractions can be used in downstream applications based on their properties, generating either high-volume and low-value or low-volume and high-value bio-based products.^11–13^

*Salicornia* is a genus of halophyte herbs belonging to the Amatanthaceae family, with species endemic to every continent except South America and Australia.^14^ The genus *Salicornia* includes 117 species, with *S. herbacea*, *S. bigelovii*, *S. europea*, *S. prostata*, *S. ramosissima*, and *S. verginica* being the most widespread. The plants are distributed extensively throughout Europe's shorelines, from the Arctic to the Mediterranean and including the Caspian and Black Sea.^15^*Salicornia dolichostachya* is a species native to European boreo-temperate biomes, where it acts as a pioneer plant in coastal areas, acquiring most of the nutrients from flooding seawater.^16^

*Salicornia* plants are rich in carbohydrates, lignin, fatty acids, proteins, as well as vitamins A, C, and E;^17,18^ whereas inorganic compounds include mainly sodium and potassium, plus other minerals.^17,19,20^ Some species of *Salicornia* are used as animal feed, and the culinary use of stems and seeds has also been described.^21,22^ Indeed, dried ground *Salicornia* plants are sold as a substitute for table salt.^23^ Some species can tolerate water with more than 1000 mM NaCl, which is higher than the average salt concentration in the oceans. Hence, these plants could be grown on a large scale and irrigated with seawater.^24,25^ Because growth in high-salinity soils is not suitable for the cultivation of other plans, *Salicornia* species could become a valuable crop in coastal areas affected by the intrusion of seawater. In Sweden, salty groundwater in continental areas may come from fossil seawater, water–rock interaction, freezing of seawater, and anthropogenic activities.^26^ Climate change may cause the sea level to rise, and hydrological cycles will lead to more areas with increased soil salinity. Because halophytes grow in areas with high salinity, and as such there is no competition with food production,^27^ their utilization as a renewable resource commands further investigation. Studies have reported yields ranging from 2.51–6.07 tons per hectare for *S. brachiata* to 35 tons per hectare for *S. bigelovii*.^28–30^

The aim of the current study was to establish an organosolv-based fractionation method for the treatment of *S. dolichostachya* fibers within a biorefinery concept. Organosolv process parameters, such as reaction temperature, treatment duration, and solvent type, can significantly affect fractionation efficiency. Here, we screened several organosolv process parameters to identify optimal conditions for maximal fractionation of *S. dolichostachya* fibers. To the best of our knowledge, no previous study on organosolv pretreatment of *Salicornia* exists, making this the first attempt towards establishing a biorefinery concept that uses *Salicornia* biomass as feedstock.

## 2. Materials and methods

### 2.1 Feedstock

*S. dolichostachya* was collected from the Wadden Sea on the Danish coast (55.307733, 8.652292). After collection, the samples were rinsed with freshwater. Using a single horizontal auger screw press (Omega, Sana, Czech Republic), two fractions were obtained: liquid juice and solid de-juiced biomass. The latter was dried in an oven at 95 °C for 24 h until constant weight was attained. The dried fibers were milled to particles smaller than 1 mm using a size reduction cutting mill (Retsch, Haan, Germany) and stored in plastic bags at room temperature until further use. The composition of the untreated biomass was 25.6% ± 1.3% w/w cellulose, 30.7% ± 1.0% w/w hemicellulose, 13.9% ± 0.1% w/w lignin, 5.3% ± 0.4% w/w ashes (partially comprised in the extractives), 9.49% ± 0.29% w/w water extractives, and 2.36% ± 0.61% w/w ethanol extractives.

### 2.2 Organosolv fractionation

**Fig. 1:**
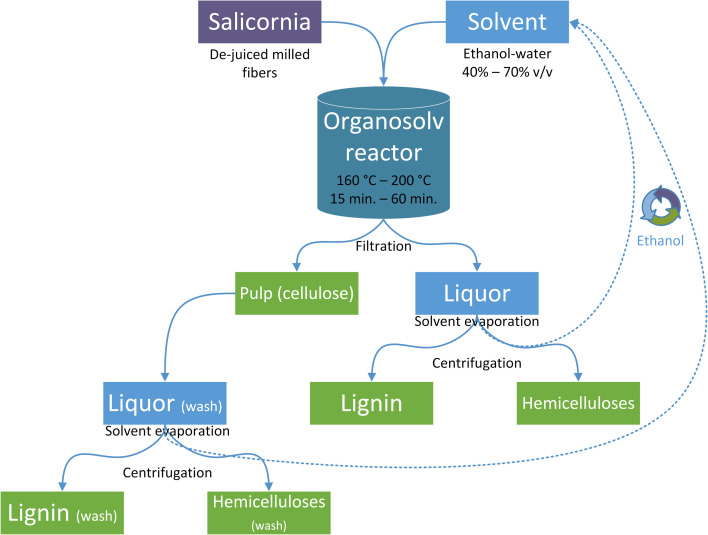
Processing of *Salicornia* biomass and recovery of cellulose, hemicellulose, and lignin fractions after organosolv pretreatment.

The milled de-juiced *S. dolichostachya* fibers were pretreated in an air-heated multidigester system comprising six 2.5-L batch autoclave reactors. For each pretreatment, 90 g dry biomass was added to a solution of ethanol : water at a 10 : 1 v/w ratio. The conditions were designed to analyze the effect of temperature (160, 180 or 200 °C), treatment time (15, 30, 45 or 60 min), and solvent composition (40%, 50%, 60% or 70% v/v) as displayed in Table 1. At the end of pretreatment, the reactors were cooled to below 40 °C and the slurry was vacuum-filtered. Next, the slurry was washed with the same solvent as above, producing an insoluble pulp fraction, liquor, and a wash phase. The latter two were processed in a rotary evaporator (Heidolph, Schwabach, Germany) to remove ethanol and precipitate lignin. The aqueous solution obtained from the filtrate and wash was centrifuged at 12 000 × *g* for 10 min at 4 °C (5804R; Eppendorf, Hamburg, Germany) to precipitate the remaining lignin, which was then added to the previously produced lignin stream, freeze-dried, and stored at room temperature. The liquid fraction containing hemicellulose-derived sugars and obtained after centrifugation was stored at 4 °C. The solid pulp was air-dried and stored in plastic bottles at room temperature. The experimental procedure is summarized in Fig. 1.

**Table 1:** Test conditions used to optimize the pretreatment of *S. dolichostachya* fibers

Variables	Code	Temperature	Time	Ethanol content
Temperature	2B6	160 °C	30 min	60% v/v
	1B6	180 °C		
	0B6	200 °C		
Time	1A6	180 °C	15 min	60% v/v
	1B6		30 min	
	1C6		45 min	
	1D6		60 min	
Ethanol content	1C4	180 °C	45 min	40% v/v
	1C5			50% v/v
	1C6			60% v/v
	1C7			70% v/v

### 2.3 Analysis

Untreated and pretreated biomass were analyzed in terms of cellulose, hemicellulose, and lignin composition according to the National Renewable Energy Laboratory protocol for the determination of structural carbohydrates and lignin in biomass.^31^ The sugar concentration was measured on a high-performance liquid chromatography (HPLC) apparatus (PerkinElmer, Waltham, MA, USA) equipped with an Aminex HPX-87H column (Bio-Rad, Hercules, CA, USA) and a refractive index detector. The column was operated at 65 °C with 5 mM H_2_SO_4_ as mobile phase at a flow rate of 0.6 mL min^−1^. Monomeric sugars present in the recovered hemicellulose fraction were determined directly by HPLC; whereas oligomeric sugars were first hydrolyzed to monomers by adding H_2_SO_4_ to a final concentration of 4% w/w and incubating at 121 °C for 1 h. Following neutralization with CaCO_3_, the resulting sugars were analyzed by HPLC. The inorganic ash content was determined gravimetrically by ashing the samples at 550 °C for 3 h, with a temperature increase of 1 °C min^−1^. Moisture content was determined gravimetrically after drying the samples at 95 °C overnight until constant weight was attained. To determine the extractives present in the untreated biomass, Soxhlet extraction was carried out first with water and then with ethanol. The solvents were evaporated in a rotary evaporator (Heidolph), and the fractions were quantified and stored. The analysis was performed in duplicates.

Size-exclusion chromatography was performed with a gel permeation column to determine the molecular weight distribution of lignins. First, acetobromination of lignins was performed by mixing 5 mg lignin with 0.9 mL glacial acetic acid and 0.1 mL acetyl bromide. Next, the mixture was stirred at 500 rpm for 2 h at room temperature in closed vials. The mixture was then transferred to round-bottom flasks in a rotary evaporator (Heidolph) and dried at 50 °C and 50 mBar. The dried material was washed two times with tetrahydrofuran, the solvent was evaporated, and the sample was solubilized in 1 mL tetrahydrofuran. After filtering through 0.22 μm hydrophobic filters, the sample was analyzed directly by HPLC using a Styragel® HR 4E column (Waters, Milford, MA, USA) and a UV detector at 280 nm. The column was operated at 40 °C, with tetrahydrofuran as mobile phase at a flow rate of 0.6 mL min^−1^. The numbers were rounded up at 100 s due to the resolution of the method.

### 2.4 Calculations

The delignification yield was calculated according to the following formula:1
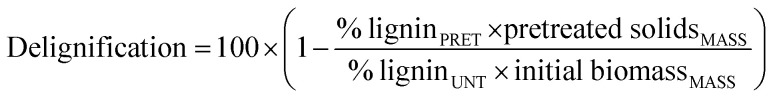
where % lignin indicates the content of lignin (w/w) in either untreated or pretreated biomass, initial biomass_MASS_ indicates the weight in grams of dry biomass used during pretreatment, and pretreated solids_MASS_ indicates the dry weight in grams of pretreated solid biomass.

Hemicellulose fractionation was calculated according to the following formula:2
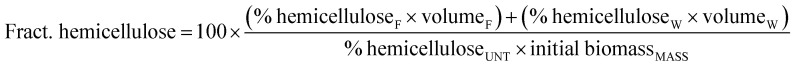
where % hemicellulose indicates the weight percentage of hemicellulose in either untreated material or pretreated (F) and wash (W) liquids, and volume indicates the liquor volume after filtration and ethanol evaporation for the filtrate (F) and wash (W) liquids.

The solubilization yield of the different fractions was calculated according to the following formula:3
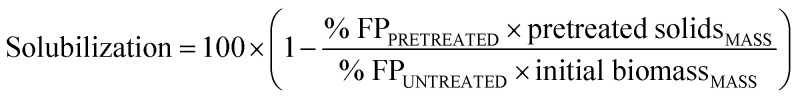
where FP indicates the fractionated product (cellulose, hemicellulose or lignin) or ashes in either pretreated or untreated solid biomass.

### 2.5 Enzymatic saccharification

Enzymatic saccharification of pretreated biomass was evaluated using the commercial cellulase enzyme solution Cellic® CTec2 (Novozymes A/S, Bagsværd, Denmark) at an enzyme load of 20 FPU g_solids_^−1^. The reactions were performed in duplicate inside 2 mL microcentrifuge tubes containing 1.0 mL solutions comprising 3% w/w dry solids in 50 mM citrate buffer (pH 5). The mixture was incubated in a thermomixer at 50 °C and 800 rpm for 72 h, with samples taken at 8, 24, 48, and 72 h. After collection, the samples were placed in a water bath at 100 °C for 5 min to denature the enzyme and then centrifuged at 12 000 × *g* for 10 min at room temperature. The supernatant was removed from the solids, filtered through a 0.22 μm syringe filter (Sartorius, Göttingen, Germany) and the sugars were quantified by HPLC as previously described (see section “Organosolv fractionation”).

## 3. Results and discussion

### 3.1 Organosolv pretreatment

#### 3.1.1 Pretreated solids fraction

Organosolv pretreatment of *S. dolichostachya* fibers was designed for the stepwise optimization of different process parameters, including temperature, time, and ethanol content (Table 1). Table 2 details the composition of pretreated solids, alongside recovery of the main biomass fractions (cellulose, hemicellulose, and lignin), and ashes under different conditions.

**Table 2:** Pretreated solids composition^a^

Code		Pretreated solids yield (% w/w)	Cellulose (% w/w)	Solub. (%)	Hemicelluloses (% w/w)	Solub. (%)	Lignin (% w/w)	Solub. (%)	Ashes (% w/w)	Solub. (%)
Temp.	2B6	64.02	31.14 ± 0.09	13.40	32.20 ± 2.08	25.60	16.56 ± 0.40	15.30	4.95 ± 0.08	33.60
	1B6	44.13	43.53 ± 1.45	16.50	22.78 ± 3.45	63.60	15.94 ± 0.59	43.80	6.38 ± 0.05	41.00
	0B6	36.72	51.27 ± 1.74	18.16	10.52 ± 0.7	86.00	14.86 ± 0.30	56.39	7.39 ± 0.10	43.10
Time	1A6	49.11	45.63 ± 0.67	2.60	20.09 ± 0.24	64.20	13.74 ± 0.64	46.10	5.62 ± 0.04	42.10
	1B6	44.13	43.53 ± 1.45	16.50	22.78 ± 3.45	63.60	15.94 ± 0.59	43.80	6.38 ± 0.05	41.00
	1C6	41.05	47.31 ± 0.35	15.60	17.06 ± 1.97	74.60	11.99 ± 1.02	60.70	6.63 ± 0.94	42.90
	1D6	40.60	45.91 ± 0.03	19.00	14.91 ± 1.56	78.10	13.70 ± 1.66	55.60	6.53 ± 0.02	44.40
Ethanol	1C4	40.06	46.83 ± 0.45	18.40	13.68 ± 0.10	80.10	16.13 ± 0.52	48.30	6.21 ± 0.53	47.80
	1C5	46.19	42.63 ± 0.28	14.40	21.56 ± 0.02	63.90	13.45 ± 0.27	50.30	5.89 ± 0.09	43.00
	1C6	40.60	45.91 ± 0.35	19.00	14.91 ± 1.97	78.10	13.70 ± 1.02	55.60	6.53 ± 0.94	44.40
	1C7	40.66	47.00 ± 0.98	16.90	9.42 ± 0.18	86.10	23.81 ± 0.40	22.60	5.57 ± 0.34	52.50
Untreated	—	—	25.56 ± 1.30	—	30.66 ± 0.97	—	13.90 ± 0.06	—	5.30 ± 0.41	—

a Codes: 0-pretreatment at 200 °C; 1-pretreatment at 180 °C; 2-pretreatment at 160 °C; A-pretreatment for 15 min; B-pretreatment for 30 min; C-pretreatment for 45 min; D-pretreatment for 60 min; 4–40% v/v ethanol content; 5–50% v/v ethanol content; 6–60% v/v ethanol content; 7–70% v/v ethanol content.

First, we tested the effect of treatment temperature (from 160 °C to 200 °C) under constant time (30 min) and ethanol content (60% v/v) on the fractionation of *Salicornia* fibers. With increasing temperature, a higher proportion of the initial biomass was solubilized, reducing the yield of pretreated solids from 64.02% to 36.72%, and boosting cellulose content from 31.14% w/w to 51.27% w/w (Table 2). The final cellulose content was 2-fold higher than in untreated *S. dolichostachya* fibers. This was matched by the proportional decrease in hemicellulose (from 32.2% w/w to 10.52% w/w) and lignin (from 16.56% w/w to 14.86% w/w) content in pretreated solids.

Increased temperature had a positive impact on delignification. A higher delignification is a desirable outcome as it creates a biomass with less lignin, which facilitates subsequent processing. Nevertheless, the higher delignification (56.39%) observed at 200 °C was not coupled to an increase in hemicellulose recovery (see “Hemicelluloses fraction” section), which could be related to sugar degradation into side-products, such as furans and organic acids (levulinic acid, formic acid, and acetic acid). Because such side-products lead to lost sugar mass and inhibit microbial growth,^32^ 180 °C was selected as the optimal temperature for further studies.

Next, we examined the effect of treatment time under constant temperature (180 °C) and ethanol content (60% v/v). A longer time promoted biomass solubilization and improved fractionation. The highest delignification rate (60.7%) was achieved with 45 min pretreatment, which was higher than pretreatment at 200 °C for 30 min (Table 2). This result demonstrated the importance of testing different parameters to select the best pretreatment conditions for the desired product. When the treatment was extended to 60 min, delignification dropped to 55.60%, resulting in more lignin being recovered from pretreated solids. This can be attributed to the formation of pseudolignin from hemicellulose decomposition,^33^ indicating that the conditions were harsh for this biomass stream. Hemicellulose removal from pretreated solids increased proportionally with the duration of pretreatment. As discussed previously, the depolymerization of hemicellulose sugars is expected to increase with a more severe pretreatment (*i.e.*, with prolonged treatment time). Hence, 45 min was chosen as the optimal treatment time for further studies.

Lastly, the effect of ethanol content was studied under stable temperature (180 °C) and treatment time (45 min). As the ethanol content was raised from 40% v/v to 60% v/v, delignification showed a marked increase, but decreased sharply when ethanol content was augmented further to 70% v/v (Table 2). Achieving an optimal ethanol : water ratio during organosolv fractionation is very important as water facilitates the hydrolysis of bonds between sugars by increasing the hydrogen ion concentration and thus lowering the pH in the solution.^34^ While ethanol promotes lignin dissolution into the liquor because of lignin's superior solubility in ethanol, the acidic conditions created by water are also necessary to cleave bonds and liberate lignin. On the one hand, the recovery of hemicellulose in pretreated solids increased as ethanol content went from 40% to 50% v/v, but dropped rapidly thereafter. On the other hand, as discussed above, lignin recovery in pretreated solids decreased as ethanol content rose to 60% v/v, but improved drastically at 70% ethanol. These findings indicated that the highest ethanol content was not optimal for the pretreatment of *Salicornia* fibers, as it failed to efficiently fractionate lignin from lignocellulosic biomass. Correlating the cellulose content in untreated fibers with pretreated solids revealed that the former improved from 66.8% to 83.9% as ethanol content went from 50% to 70% v/v, respectively.

#### 3.1.2 Lignin fraction

Lignin samples isolated under different organosolv process parameters were analyzed for impurities, such as sugars and ashes, as well as Klasson lignin content (Table 3). Overall, the purity of the obtained lignins was very high, with only two samples (180 °C for 15 min with 60% ethanol and 180 °C for 45 min with 50% ethanol) exceeding 6% sugar contamination, which indicated suitable fractionation during pretreatment. Sugar contamination of only 1.22% w/w was obtained during pretreatment at 200 °C. The ashes present in lignin after organosolv pretreatment did not exceed 1.8% w/w, with most samples exhibiting less than 1% w/w ashes content. Altogether, the few impurities found in lignin pointed to highly efficient organosolv fractionation of biomass.

**Table 3:** Lignin fractions composition and molecular weight^a^

Code		Cellulose (% w/w)	Hemicelluloses (% w/w)	Klasson lignin (% w/w)	Ashes (% w/w)	*M*_N_ (g mol^−1^)	*M*_w_ (g mol^−1^)	DI
Temp.	2B6	0.18 ± 0.02	1.72 ± 0.99	83.68 ± 0.90	1.08 ± 0.05	600	1800	3.00
	1B6	0.19 ± 0.02	4.59 ± 0.34	84.96 ± 2.98	1.25 ± 0.07	600	1100	1.83
	0B6	0.46 ± 0.04	0.76 ± 0.09	84.66 ± 2.12	0.53 ± 0.07	600	1200	2.00
Time	1A6	3.39 ± 0.32	7.01 ± 0.54	72.91 ± 2.18	1.79 ± 0.06	600	1200	2.00
	1B6	0.19 ± 0.02	4.59 ± 0.34	84.96 ± 2.98	1.25 ± 0.07	600	1100	1.83
	1C6	0.00 ± 0.00	2.87 ± 0.28	86.78 ± 0.77	0.68 ± 0.14	700	1600	2.29
	1D6	1.36 ± 0.08	2.66 ± 0.19	90.20 ± 0.53	0.91 ± 0.05	600	1300	2.17
Ethanol	1C4	2.01 ± 0.02	3.50 ± 0.18	77.91 ± 6.31	0.86 ± 0.02	600	1200	2.00
	1C5	2.51 ± 0.37	4.63 ± 0.81	81.98 ± 1.47	0.95 ± 0.07	700	1900	2.71
	1C6	0.00 ± 0.00	2.87 ± 0.28	86.78 ± 0.77	0.68 ± 0.14	700	1600	2.29
	1C7	1.63 ± 0.14	3.91 ± 0.33	81.04 ± 0.95	0.96 ± 0.03	600	1500	2.50

a *M*_N_: number average; *M*_w_: weight average; DI: dispersity index (*M*_W_/*M*_N_). Codes: 0-pretreatment at 200 °C; 1-pretreatment at 180 °C; 2-pretreatment at 160 °C; A-pretreatment for 15 min; B-pretreatment for 30 min; C-pretreatment for 45 min; D-pretreatment for 60 min; 4–40% v/v ethanol content; 5–50% v/v ethanol content; 6–60% v/v ethanol content; 7–70% v/v ethanol content.

Size-exclusion chromatography was performed for lignin samples of *S. dolichostachya* to determine their molecular weight distribution. Overall, the different pretreatments yielded a number average molecular weight ranging from 600 Da to 700 Da, while the weight average was between 1100 Da and 1900 Da (Table 3). Dispersity was highest (3.00) at 160 °C for 30 min with 60% ethanol, which coincided with lower delignification and indicated non-extensive depolymerization of lignin. In our previous study with birch (*Betula pendula* L.) sawdust, the *M*_w_ ranged from 1800 Da (180 °C for 15 min with 50% v/v ethanol) to 15 900 Da (180 °C for 30 min with 60% v/v ethanol).^35^ When using birch chips, the *M*_w_ ranged from 2700 Da (200 °C for 15 min with 60% ethanol and 1% w/w biomass H_2_SO_4_) to 8000 Da (200 °C for 30 min with 60% ethanol without catalyst).^36^ Pine wood pretreated at 190 °C for 60 min with 60% v/v ethanol and 1% w/w biomass H_2_SO_4_ generated lignins with *M*_w_ of 7700 Da; whereas cotton stalks pretreated at 200 °C for 45 min with 50% v/v ethanol and 1% w/w biomass H_2_SO_4_, as well as sweet sorghum bagasse pretreated at 180 °C for 30 min with 60% v/v ethanol produced lignins with *M*_w_ of 16 800 Da and 6600 Da, respectively.^37^

Comparatively, organosolv-pretreated lignin isolated from *Salicornia* had a much smaller molecular weight, which can be explained by the catalytic action of transition metals (*e.g.*, iron, cobalt, manganese, platinum, ruthenium, and rhodium) during oxidative cleavage of β-O-4 linkages.^38–40^ The conversion of lignin into downstream compounds requires homogeneity of the starting material, which can be a challenge in the case of complex fragmented lignin precursors.^41^ In this context, low molecular weight lignin is more advantageous as it is more prone to depolymerization, which facilitates its valorization. A plethora of value-added products can be synthesized from low molecular weight lignin, namely vanillin, bioplastic, pigments, resins, dyes, biodiesel, and polymers.^40,42^

#### 3.1.3 Hemicelluloses fraction

Fractionated hemicellulose under different organosolv process parameters was analyzed with respect to sugar composition (*e.g.*, monomers and oligomers) and sugar origin (*e.g.*, cellulose or hemicellulose) (Table 4).

**Table 4:** Recovery of hemicelluloses as a separate fraction^a^

Code		Oligomers (% w/w)	Fractionated hemicellulose (% w/w)	Monomers (g/100 g_biomass_)			Oligomers (g/100 g_biomass_)			Total cellulose sugars (g/100 g_biomass_)	Total hemicel sugars (g/100 g_biomass_)
				Glucose	Hemicel.	Total	Glucan	Hemicel.	Total		
Temperature	2B6	69.5 ± 3.0	17.0 ± 1.6	0.01 ± 0.00	1.61 ± 0.36	1.63 ± 0.36	0.10 ± 0.01	3.61 ± 0.45	3.71 ± 0.45	0.11 ± 0.01	5.22 ± 0.58
	1B6	69.0 ± 0.9	28.9 ± 0.8	0.00 ± 0.00	2.82 ± 0.62	2.82 ± 0.62	0.16 ± 0.00	6.11 ± 0.12	6.27 ± 0.12	0.16 ± 0.00	8.93 ± 0.63
	0B6	37.8 ± 3.0	19.2 ± 0.9	0.07 ± 0.01	3.79 ± 0.76	3.87 ± 0.76	0.21 ± 0.02	2.14 ± 0.19	2.35 ± 0.20	0.28 ± 0.03	5.94 ± 0.78
Time	1A6	70.0 ± 2.0	18.3 ± 1.2	0.11 ± 0.03	1.72 ± 0.41	1.83 ± 0.41	0.11 ± 0.03	4.16 ± 0.01	4.27 ± 0.04	0.21 ± 0.04	5.89 ± 0.41
	1B6	69.0 ± 0.9	28.9 ± 0.8	0.00 ± 0.00	2.82 ± 0.62	2.82 ± 0.62	0.16 ± 0.00	6.11 ± 0.12	6.27 ± 0.12	0.16 ± 0.00	8.93 ± 0.63
	1C6	69.0 ± 2.3	25.2 ± 2.3	0.08 ± 0.01	2.54 ± 0.60	2.62 ± 0.60	0.41 ± 0.02	5.41 ± 0.20	5.82 ± 0.20	0.49 ± 0.02	7.95 ± 0.63
	1D6	55.4 ± 8.1	20.9 ± 3.6	0.12 ± 0.03	2.94 ± 0.81	3.06 ± 0.81	0.35 ± 0.06	3.44 ± 1.02	3.80 ± 1.02	0.48 ± 0.06	6.39 ± 1.30
Ethanol	1C4	51.3 ± 0.5	29.0 ± 0.3	0.13 ± 0.01	4.56 ± 1.10	4.69 ± 1.10	0.52 ± 0.00	4.41 ± 0.02	4.94 ± 0.02	0.66 ± 0.01	8.97 ± 1.10
	1C5	55.8 ± 0.7	29.1 ± 0.4	0.25 ± 0.06	3.98 ± 0.92	4.23 ± 0.92	0.33 ± 0.00	5.00 ± 0.05	5.34 ± 0.05	0.58 ± 0.06	8.98 ± 0.92
	1C6	69.0 ± 2.3	25.2 ± 2.3	0.08 ± 0.01	2.54 ± 0.60	2.62 ± 0.60	0.41 ± 0.02	5.41 ± 0.20	5.82 ± 0.20	0.49 ± 0.02	7.95 ± 0.63
	1C7	41.9 ± 4.0	23.9 ± 1.6	0.22 ± 0.04	4.36 ± 0.83	4.58 ± 0.83	0.30 ± 0.02	2.99 ± 0.41	3.30 ± 0.41	0.52 ± 0.05	7.35 ± 0.92

a Codes: 0-pretreatment at 200 °C; 1-pretreatment at 180 °C; 2-pretreatment at 160 °C; A-pretreatment for 15 min; B-pretreatment for 30 min; C-pretreatment for 45 min; D-pretreatment for 60 min; 4–40% v/v ethanol content; 5–50% v/v ethanol content; 6–60% v/v ethanol content; 7–70% v/v ethanol content.

The temperature of 180 °C was optimal for the fractionation of hemicellulose as it allowed for the highest percentage of oligomers (69.0%) and hemicellulosic sugars (8.93 g/100 g_biomass_) to be recovered. On the one hand, a higher temperature during pretreatment led to fewer oligomers (37.8%), because the harsh conditions (200 °C) favored the hydrolysis of hemicellulosic sugars. On the other hand, a lower temperature (160 °C) was not optimal for the solubilization of hemicelluloses (only 5.22 g/100 g_biomass_) due to lower fractionation of biomass during pretreatment (Table 2). These findings confirmed how the increased severity of pretreatment promoted hemicellulose depolymerization into monomers.^43^

During pretreatment, hemicellulosic sugars are released within the fractionated biomass and then hydrolyzed into monomeric sugars. Hence, the duration of pretreatment is a decisive factor as it needs to balance delignification with hydrolysis, while minimizing the conversion of sugars into secondary products.^44^ Hemicellulose fractionation was optimal at 30 min pretreatment, with yields dropping after 45 min of pretreatment and the percentage of oligomers after 60 min (Table 4).

As explained previously (see “Pretreated solids fraction”), excessive ethanol content lowers biomass hydrolysis, leading to lower delignification and fractionation of hemicellulose. This was observed in our study, where a similar fractionation of hemicellulose (∼29%) was observed for 40% and 50% v/v ethanol, followed by consistent reduction (to 25.2% and 23.9%) with increased ethanol concentration. While the oligomer to monomer ratio tended to increase with increasing ethanol content, it dropped dramatically at 70% v/v ethanol, suggesting a more pronounced hydrolysis of oligomers to monomers at this point. A higher yield of oligomers is desired for the use of hemicellulose in prebiotics, feed, food packaging, and food ingredients. Because enzymatic hydrolysis of oligomers into monomers can be easily achieved downstream, lower hydrolysis of hemicelluloses during fractionation is preferred.^45^ Taking into account the above, treatment with 50% v/v ethanol seems to be the most favorable as it results to the highest hemicellulosic sugar production (8.98 g/100 g_biomass_), alongside the highest fractionated hemicellulose (29.1% w/w) and the second highest oligomer ration (55.8% w/w).

Cybulska *et al.* (2013) studied the effects of hydrothermal pretreatment on *S. bigelovii* at three different temperatures (190, 200, and 210 °C). They observed a progressive reduction in the recovery of xylose, in both pretreated solids and liquid, accompanied by a concomitant increase in furfural. This finding highlighted the degradation of pentose sugars with increased time and consequent severity of pretreatment.^32,46^

### 3.2 Enzymatic saccharification

**Fig. 2:**
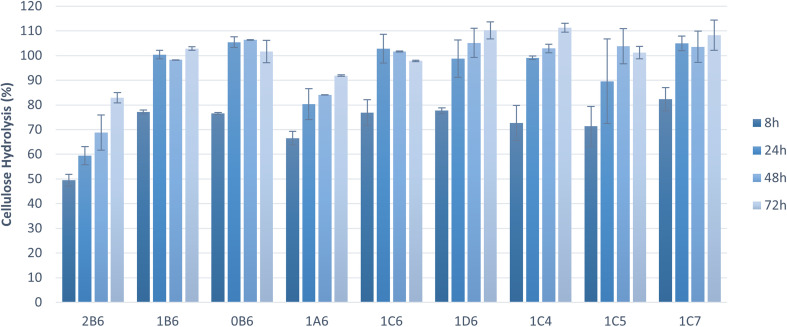
Cellulose hydrolysis yields of *S. dolichostachya* pretreated biomass samples. Codes: 0-pretreatment at 200 °C; 1-pretreatment at 180 °C; 2-pretreatment at 160 °C; A-pretreatment for 15 min; B-pretreatment for 30 min; C-pretreatment for 45 min; D-pretreatment for 60 min; 4–40% v/v ethanol content; 5–50% v/v ethanol content; 6–60% v/v ethanol content; 7–70% v/v ethanol content.

**Fig. 3:**
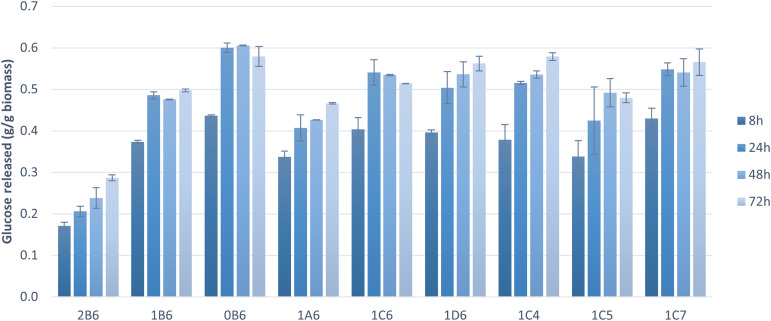
Glucose release during enzymatic saccharification in *S. dolichostachya* pretreated biomass samples. Codes: 0-pretreatment at 200 °C; 1-pretreatment at 180 °C; 2-pretreatment at 160 °C; A-pretreatment for 15 min; B-pretreatment for 30 min; C-pretreatment for 45 min; D-pretreatment for 60 min; 4–40% v/v ethanol content; 5–50% v/v ethanol content; 6–60% v/v ethanol content; 7–70% v/v ethanol content.

To assess the suitability of pretreated solids as feedstock for microbial cultivations, the pretreated pulp fractions were subjected to enzymatic saccharification. The hydrolysis of cellulose to glucose was near complete (100%) within 72 h in the majority of tested samples, and within 24 h in six of these samples (Fig. 2). Saccharification yields were high (>70%) also at the initial stage of saccharification (8 h). Pretreatment parameters affected the saccharification results. Both pretreatments at higher temperatures (180 °C and 200 °C) allowed significantly higher saccharification within 8 h and complete saccharification within 24 h whereas the one at 160 °C resulted in a much lower saccharification rate, likely due to lower delignification and fractionation of hemicellulose (Table 3). These factors hindered the enzymes' action and led to longer incubation times to achieve better cellulose hydrolysis yields, as observed by an increase in the release of glucose towards 72 h (Fig. 2 and 3). A treatment of 15 min resulted in low cellulose hydrolysis after 8 h of saccharification. The result improved with a pretreatment of 30 min but did not change any further thereafter. Complete saccharification was achieved with all treatment times except 15 min (Fig. 2), which can be attributed to lower delignification and removal of hemicellulose (Table 2). Finally, ethanol content of up to 50% did not have any impact on the results obtained at 8 h, and only 70% v/v ethanol caused a marginal improvement. In all cases, complete saccharification was achieved when the reactions were extended to 24 h. Cybulska *et al.* (2013) reported yields of 87–92% following the saccharification of hydrothermally pretreated *S. bigelovii* biomass.^46^ Smichi and collaborators (2018) evaluated organosolv pretreatment of the halophyte Juncus maritimus with H_3_PO_4_ at 50 °C for 24 h. Saccharification of the resulting cellulosic pulp reached a 90% hydrolysis yield after 48 h using the same enzyme as in the present study with an initial load of 61.25 cm^3^ U mL^−1^.^47^ Taken together, these findings indicate that organosolv pretreatment is an outstanding method for processing halophytes, enabling elevated cellulose saccharification due to increased accessibility of the fractionated feedstock to the hydrolyzing enzymes.

Cellulose hydrolysis yield is a very important factor when assessing the suitability of pretreated solids for microbial conversion processes. However, owing to differences in cellulose content, it is sometimes more informative to consider the amount of glucose released per gram of solids. Because most samples achieved total saccharification (Fig. 2), the total release of glucose relative to the initial biomass was calculated (Fig. 3). Pretreatment at 200 °C achieved complete cellulose hydrolysis as the majority of the pretreatments tested but, due to the higher amount of cellulose present in this sample (Table 2), it released the highest amount of glucose (0.61 g g_biomass_^−1^). The sample pretreated at 160 °C exhibited the lowest release of glucose during saccharification and the lowest cellulose content, which can be attributed to its lower delignification and fractionation of hemicellulose (Table 3). These findings highlight the tight link between saccharification and proper biomass fractionation. Larran and collaborators (2015) studied the saccharification of the halophyte *Spartina argentinensis* following pretreatment with laccase. Using 0.4 U of commercial enzymes, they achieved the release of 0.035 g g_biomass_^−1^ of glucose within 24 h.^48^ Accordingly, it can be concluded that the majority of pretreated solids are amenable to anaerobic digestion, but the choice of a suitable organosolv pretreatment will strongly affect the process.

## 4. Conclusions

The present study demonstrated that *Salicornia* fibers served as an excellent substrate for organosolv fractionation, achieving purified fractions of cellulose, hemicellulose, and lignin. Different process parameters, including pretreatment temperature, duration, and solvent content, were tested. The highest tested temperature achieved excellent delignification, but at the expense of hemicellulose recovery, particularly in the form of oligomers. Taking into account total biomass recovery and hemicellulose yield, the optimal treatment temperature appeared to be 180 °C. When assessing the duration of pretreatment, delignification, cellulose content, and fractionation of hemicellulose were optimal at 45 min, with the proportion of hemicellulose oligomers decreasing following longer pretreatments. Delignification and the oligomers ratio were optimal with 60% ethanol, whereby cellulose content in biomass increased by 79.6% compared to untreated fibers. Finally, enzymatic saccharification trials demonstrated that cellulose from pretreated solids was easily hydrolysable to glucose and in most cases complete conversion of cellulose was attained. In summary, we demonstrate that *S. dolichostachya* fibers can be used as a novel sustainable feedstock for biomass biorefineries, thereby widening the portfolio of renewable biomass sources.

## Conflicts of interest

There are no conflicts to declare.

## Supplementary Material
